# Small breast epithelial mucin tumor tissue expression is associated with increased risk of recurrence and death in triple-negative breast cancer patients

**DOI:** 10.1186/1746-1596-8-71

**Published:** 2013-05-01

**Authors:** Liang Liu, Zhaozhe Liu, Shuxian Qu, Zhendong Zheng, Yongye Liu, Xiaodong Xie, Fulin Song

**Affiliations:** 1 Department of Oncology, Cancer Center of General Hospital of Shenyang Military Region, Shenyang 110840, China; 2 LiCina; 3 Department of Oncology, Weifang People's Hospital, Weifang, 261041, China; 4 Department of Pathology, General Hospital of Shenyang Military Region, Shenyang, 110840, China

**Keywords:** SBEM, DFS, OS, IHC, TNBC

## Abstract

**Background:**

Small breast epithelial mucin (SBEM) has been implicated in tumor genesis and micrometastasis in breast cancer. Triple-negative breast cancer (TNBC) was characterized by high incidence in young women,early relapse and a very poor prognosis. The aim of this study was to evaluate the association of SBEM expression in tissues of TNBC with disease-free survival (DFS) and overall survival (OS).

**Methods:**

SBEM protein expression was detected in 87 available formalin-fixed paraffin-embedded (FFPE) tissue specimens from TNBC patients by means of immunohistochemistry (IHC). We analyzed the correlation between the SBEM protein expression and DFS and OS during a 5 year follow-up period, respectively. And a SBEM cut-off value of prognosis was established associated with DFS and OS. SBEM was analyzed against other risk factors in multivariate analysis.

**Results:**

SBEM 3+ score was cut-off value of prognosis and significantly correlated with DFS (*p* = 0.000) and OS (*p* = 0.001) in TNBC patients. There was a marked associations (*p* <0.05) between SBEM 3+ score and tumor size, grade, node status, TNM stage and Ki67. Multivariate analysis showed that patients with SBEM 3+ represented a higher risk of recurrence and mortality than those with a lower SBEM expression (HR = 3.370 with *p* = 0.008 for DFS and HR = 4.185 with *p* = 0.004 for OS).

**Conclusions:**

SBEM is an independent risk predictor and may offer utility as a prognostic marker in TNBC patients.

**Virtual Slides:**

http://www.diagnosticpathology.diagnomx.eu/vs/1624613061936917

## Introduction

Breast cancer remains to be an important public health problem. Triple-negative breast cancer (TNBC), one of five molecular subtypes recognized in 2000 [1,2], lacks estrogen receptor (ER), progesterone receptor (PR) and human epidermal growth factor receptor 2 (HER2) expression. TNBC is characterized by high incidence in young women, early recurrence and shows a relative sensitivity of chemotherapy. Most studies [3-5] showed that the prognosis of TNBC was less favorable than that of non- TNBC. Therefore, more attention is being paid to unraveling the oncogenes that expressing at inappropriately high levels or being altered to have novel properties, which leads to invasion and metastasis of carcinoma cells [6]. Small breast epithelial mucin (SBEM) is a newly cloned gene and expresses in breast cancer cell lines rather than in cell lines of non-breast origin [7]. SBEM was only expressed in mammary and salivary glands [7]. High SBEM expression was found to be strongly associated with the histopathological detection of lymph node metastases [7]. Several laboratories showed that SBEM expression correlated with higher tumor grade [8], TNM staging and lymph node metastasis at both mRNA and protein levels [9]. SBEM protein was more frequently observed in ER- than in ER + breast cancers [8,10] and SBEM expression showed a trend towards an association with decreased OS and DFS in SBEM + patients [8]. Valladares Ayerbes et al. [10] studied the expression profiles of SBEM gene in silico and in vitro, and demonstrated that SBEM-mRNA could serve as a marker for bone marrow micro metastasis in breast cancer patients [10]. Researchers also reported that SBEM had the potential to be a specific marker for predicting hematogenous micro metastasis and responses to neo-adjuvant chemotherapy in breast cancer [9].

Based on the above information, SBEM might play an important role in progression and metastasis of breast cancer, especially in TNBC. The aim of this study was to analyze association of SBEM expression in tissue of TNBC with clinical- pathological features, DFS and OS, and to identify new prognostic and/or predictive biomarkers for TNBC patients.

## Materials and methods

The study examined cases from 126 patients diagnosed between 2006 and 2008 in our hospital. 39 cases without evaluable tumor tissue were excluded from the analysis. The final database for analysis included 87 cases with histological confirmation. Clinical data of all the cases were reviewed retrospectively from medical records in our hospital. All patients were females and had a minimum 5 years’ follow-up records. All the patients underwent operational treatment according to clinical practice guidelines of National Comprehensive Cancer Network (NCCN) of the United States. None of the patients received neo-adjuvant therapy. Statistic and analysis of clinicopathological parameters, including age at diagnosis, disease stage, tumor size, tumor grade, lymph node status and Ki67, were listed in Table 1.

**Table 1:** Clinicopathological characteristics of patients

**Parameter**	**Number (n)**	**Subgroup cut-offs**	**SBEM < 3 score**		**SBEM = 3 score**		***P*-value**
			**Number (n)**	**%**	**Number (n)**	**%**	
Age	87		76	72.4	11	63.6	0.809
		X > 35	55	27.6	7	36.4	
		X ≤ 35	21		4		
TNM staging	87		76		11		0.047
		I (1)	36	47.4	2	18.2	
		II (2)	29	38.2	4	36.4	
		III (3)	11	14.5	5	45.5	
P53	49		40		9		0.435
		Mutated	21	52.5	7	75	
		No-mutated	19	47.5	2	25	
Node	87		76		11		0.034
		+	26	34.2	8	72.7	
		_	50	65.8	3	27.3	
Grade	67		57		9		0.471
		Low (1)	20	35.1	2	22.2	
		Mod (2)	23	40.4	3	33.3	
		High (3)	14	24.6	4	44.4	
Size	87		76		11		0.494
		X > 20 mm	40	52.6	7	63.6	
		X ≤ 20 mm	36	47.4	4	36.4	
Ki67	71		62		9		0.027
		X > 35	19	32.8	7	77.8	
		X ≤ 35	39	67.2	2	22.2	

All cases examined were ER and PR negative by IHC. HER2 status was considered negative if the immunohistochemical score was 0 or 1+, or if the score was 2+ but non-amplification by fluorescence in situ hybridization (FISH), and positive if the score was 3 +.

### SBEM expression and evaluation of IHC

All tissues were collected surgically under the supervision of an experienced pathologist. SBEM expression was measured by IHC on FFPE samples.

Streptavidinperosidase (S-P) IHC staining was performed using SBEM antibody of mouse monoclonal (diluted 1/800, Abcam plc. Cambridge, UK). The detailed procedures were done as described by Jennbacken [11].

PBS was used to replace the primary antibody in negative controls. SBEM was a secreted protein and it was mainly located in cell membrane, subordinately in cytoplasm. Normal breast tissues were in general weakly or negative. So it was evaluated and scored if cell membrane and/or cytoplasm reactivity were observed [8]. But there was no relevant clinical cut-off point and the standard evaluation methods reported for SBEM in the literature. According to our data and TMA IHC grading method by Serrero G [12] and Pan [13], our scoring was semiquantitatively categorized as: ≤5% of tumor cells staining with/without weakly stained was negative (0), followed by a score of 1 (>5% of tumor cells and with weak/focal positive staining or ≤5% of tumor cells with strongly stained), 2 (>5% of tumor cells and with moderate/focal positive staining), 3 (>5% of tumor cells and with strong/diffuse positive staining).

### Statistical analysis

The correlation between SBEM, Clinicopathological characteristics and survival outcomes was compared by Pearson’s Χ^2^ test. Survival analyses, including DFS and OS, were performed with the log rank test and all results were displayed in Kaplan–Meier. DFS was defined as the time interval from date of diagnosis to the time of last disease-free follow-up or at death for those patients who died without a previous recurrence. OS was defined as the time interval from date of diagnosis to time of last follow-up or death [14]. Time to recurrence (local, regional and distant) was censored at time of last disease-free follow-up, and at death for those patients who died without a previous recurrence [14]. Statistical significance was defined as *P* value < 0.05. SPSS17.0 software package was used for all statistical analyses.

In order to observe whether the SBEM expression had an independent prognostic value with conventional risk factors, the risk factor alone, or along with SBEM was analyzed with Cox’s proportional hazards models. Multivariate analysis was performed to determine the association of SBEM with all combined clinical risk factors on DFS and OS.

## Results

**Figure 1:**
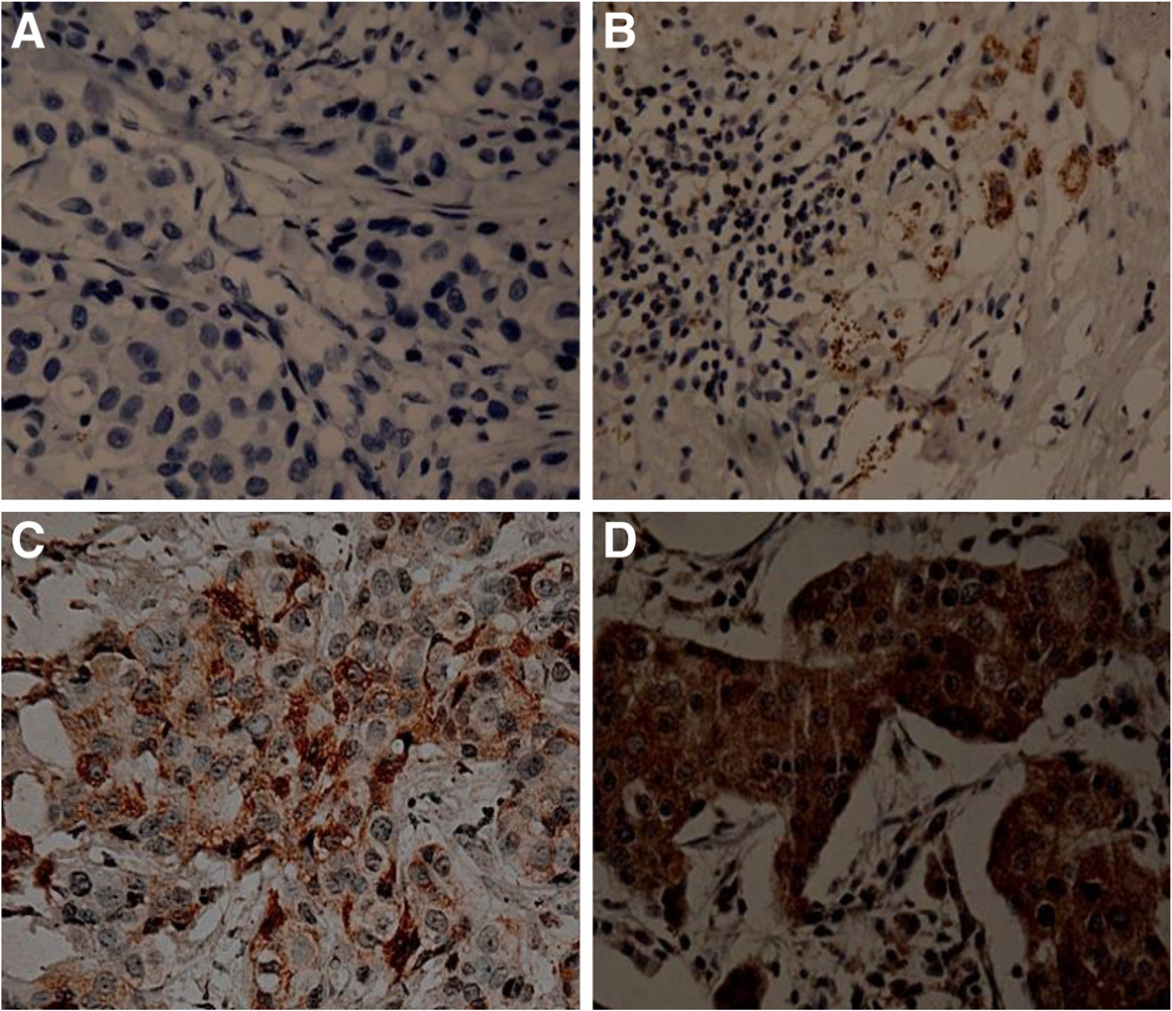
**Immunohistochemical staining for SBEM. A**: 0+: ≤5% of tumor cells staining with/without weakly stained was negative (original magnification × 200); **B**: 1+: >5% of tumor cells and with weak/focal positive staining or ≤5% of tumor cells with strongly stained (original magnification × 100); **C**: 2+: >5% of tumor cells and with moderate/focal positive staining (original magnification × 200); **D**: 3+: >5% of tumor cells and with strong/diffuse positive staining (original magnification × 200).

The clinicopathological characteristics of patients were described in Table 1. In the whole group, the median age was 42 (from 24 to 67) years old, the median DFS was 23 months (from 2 to 54 months) and median OS was 34 months (from 6 to 60 months). Figure 1 showed photomicrograph examples of SBEM expression with different scores in TNBC tissues determined by IHC.

**Figure 2:**
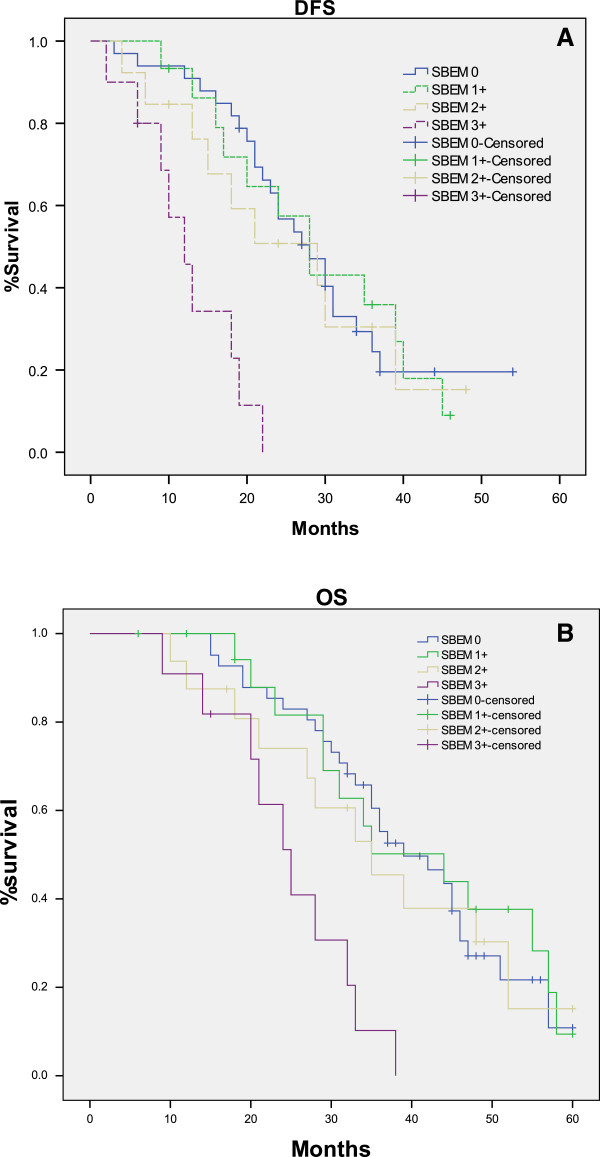
**Kaplan-Meier estimates for DFS and OS by SBEM scores.** No significant difference was found between different SBEM score (0, 1+ and 2+) and DFS (**A**) or OS (**B**) (*p* >0.05). But, there was a marked difference between SBEM 3+ score and SBEM score of 0, 1+ and 2+ (*p* <0.05) on DFS (**A**) and OS (**B**).

To evaluate SBEM prognostic significance, we analyzed SBEM score (0, 1+, 2+, and 3+) in relation with DFS and OS in TNBC patients. No significant difference was found between DFS or OS and each group (SBEM score of 0, 1+ and 2+) by pairwise comparison methods (*p* >0.05). But, there was a marked associations between SBEM 3+ score and SBEM score of 0, 1+ and 2+ (*p* <0.05) (Figure 2). The results of log-rank testing for SBEM different scores were showed in Table 2.

**Table 2:** Log-rank testing for SBEM different scores

**SBEM scores**	**DFS**		**OS**	
	**Chi-squared**	***P *Value**	**Chi-squared**	***P *Value**
**0 and 1**	**0.020**	**0.887**	**0.124**	**0.724**
**0 and 2**	**0.147**	**0.701**	**0.160**	**0.689**
**0 and 3**	**22.641**	**0.000**	**15.472**	**0.000**
**1 and 2**	**0.087**	**0.768**	**0.623**	**0.430**
**1 and 3**	**12.501**	**0.000**	**9.431**	**0.002**
**2 and 3**	**6.578**	**0.010**	**5.512**	**0.019**

**Figure 3:**
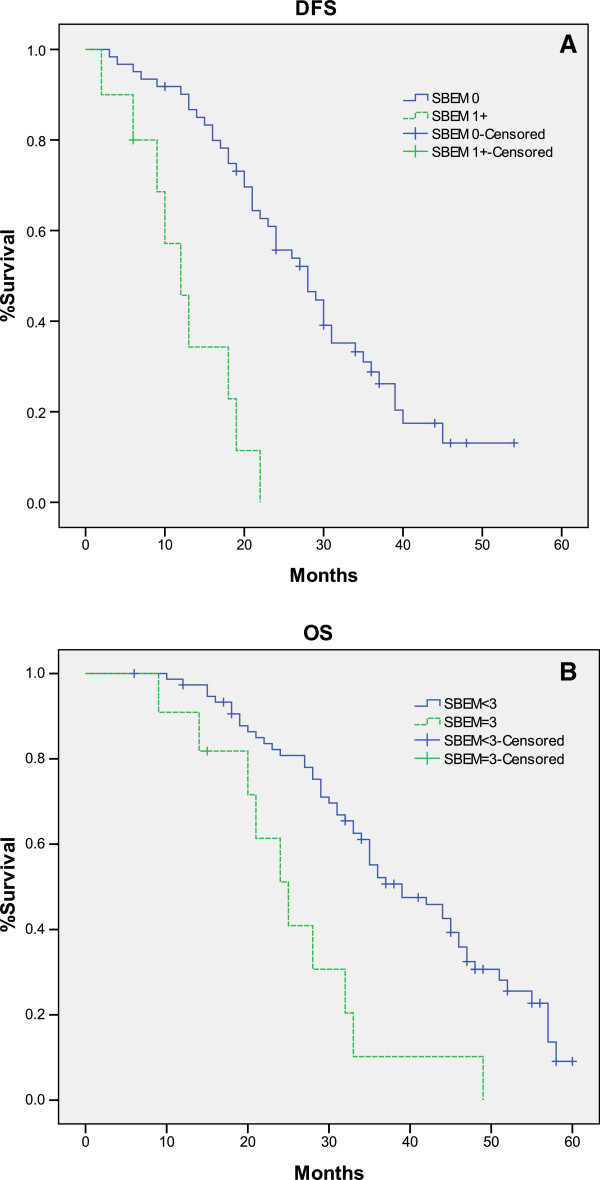
**Kaplan-Meier estimates for DFS and OS by SBEM < 3+ group and SBEM = 3+ group.** There was a significant difference between SBEM =3 + group and SBEM score <3+ group on DFS (**A**) and OS (**B**) (*p* = 0.000, *p* = 0.001, respectively).

We observed that high SBEM expression with SBEM 3+ score was consistent with high recurrence and death rates, while lower SBEM expression (0, 1+ and 2+) was reversed. Based on the statistics above, we believed that SBEM expression with SBEM 3+ score might be the SBEM cut-off value of prognosis. We divided the cases into two groups, one is the SBEM < 3+ group, the other is SBEM = 3+ group. From Figure 3, we found that DFS and OS function curves showed the large separation between SBEM < 3+ group and SBEM = 3+ group. The log-rank tests confirmed that SBEM score of 3+ was significant associated with DFS and OS (*p* = 0.000, *p* = 0.001, respectively). The results of log-rank testing for SBEM score cut-off were showed in Table 3. According to these results, the threshold effect of SBEM score 3+ was verified.

**Table 3:** Log-rank testing for SBEM score cut-off establishment

**SBEM scores**	**DFS**		**OS**	
	**Chi-squared**	***P *Value**	**Chi-squared**	***P *Value**
**0 and 1**	**0.020**	**0.887**	**0.124**	**0.724**
**0,1 and 2**	**0.120**	**0.729**	**0.399**	**0.528**
**0,1,2 and 3**	**23.524**	**0.000**	**11.595**	**0.001**

On the basis of the cut-off established, 87 patients were divided into 2 groups. Table 1 showed that there were significant associations between SBEM 3+ score and nodal involvement, TNM stage and Ki67 (*p* < 0.05). Neither SBEM < 3+ group nor SBEM = 3+ group, SBEM expression indicated no significant correlations with age, tumor size and grade. Based on the Kaplan Meier curves for DFS and OS function, the median DFS and OS of SBEM < 3+ group were 28 and 39 months, respectively, while those of SBEM = 3+ group were only 12 and 25 months, respectively. Patients with high SBEM expression had poor clinical outcomes. In SBEM = 3+ group, no patients could survive over 5 years. The longest time of OS was 38 months. In comparison, the patients in SBEM < 3+ group had the higher survival probabilities and longer OS than those in SBEM = 3+ group. We inferred that patients in SBEM < 3+ group had a higher risk of recurrence or death than those in SBEM < 3+ group.

**Figure 4:**
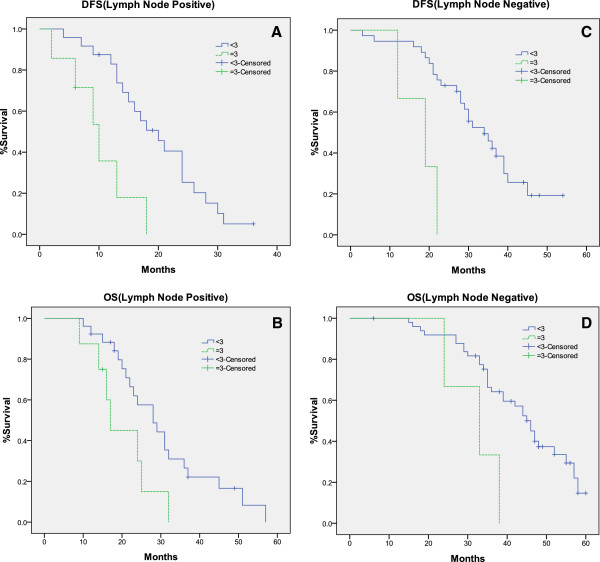
**Kaplan-Meier estimates for DFS and OS in lymph node positive and negative group.** SBEM score of 3+ was significant associated with DFS and OS in both lymph node positive (**A**, **B**) and negative group (**C**, **D**) (p <0.05).

Similar observations of Kaplan Meier curves for DFS and OS function were obtained when the cases were segregated into lymph node negative and positive group (Figure 4). The results of log-rank testing for lymph node positive group and negative group were showed in Table 4.

**Table 4:** Log-rank testing for lymph node positive group and lymph node negative group

**Lymph node**	**DFS**		**OS**	
	**Chi-squared**	***P *Value**	**Chi-squared**	***P *Value**
**positive**	**9.392**	**0.002**	**4.525**	**0.033**
**negative**	**9.960**	**0.002**	**5.524**	**0.019**

Univariate analysis showed that SBEM 3+ scores were associated with an HR of 5.768 for DFS and 4.113 for OS in Table 5 and Table 6. It was demonstrated that grade, age, disease stage, lymph node status and Ki67 for DFS and OS were other predictors of survival. From Table 5, we found that there was no marginally change when SBEM was added to each covariate in DFS. Similar results were showed for OS in Table 6.

**Table 5:** The impact of SBEM and other risk factors on DFS by Cox’s proportional hazards analysis

**Covariate**	***P *value**		**Hazard radio**		**95% Confidence interval**	
	**Univariate**	**Added SBEM**	**Univariate**	**Added SBEM**	**Univariate**	**Added SBEM**
SBEM (3+ *vs.* <3+)	0.000	0.000	5.768	5.768	2.584-12.876	2.584-12.876
Lymph Node (Pos. *vs.* Neg.)	0.000	0.000	3.589	3.433	1.996-6.451	1.893-6.228
Age (>35Y *vs.* ≤35Y)	0.749	0.785	1.103	0.918	0.606-2.006	0.495-1.702
Tumor size (>20 mm *vs.* ≤20 mm)	0.457	0.583	0.814	0.858	0.473-1.400	0.498-1.480
Grade (>1 *vs.* =1)	0.035	0.053	2.052	1.911	1.053-3.999	0.991-3.799
Grade (=3 *vs.* ≤2)	0.000	0.001	3.930	3.549	1.905-8.110	1.699-7.413
TNM (>1 *vs.* =1)	0.000	0.000	3.439	3.148	1.925-6.143	1.816-5.459
TNM (=3 *vs.* ≤2)	0.000	0.000	5.647	5.446	2.601-12.264	2.401-12.352
Ki67 (≤35 *vs.* >35)	0.001	0.004	3.060	2.714	1.576-5.942	1.366-5.393

**Table 6:** The impact of SBEM and other risk factors on OS by Cox’s proportional hazards analysis

**Covariate**	***P *value**		**Hazard radio**		**95% Confidence interval**	
	**Univariate**	**Added SBEM**	**Univariate**	**Added SBEM**	**Univariate**	**Added SBEM**
SBEM (3+ *vs.* <3+)	0.000	0.000	4.113	4.113	2.004-8.440	2.004-8.440
Lymph Node (Pos. *vs.* Neg.)	0.000	0.000	2.972	2.734	1.777-4.970	1.617-4.623
Age (>35Y *vs.* ≤35Y)	0.646	0.942	1.140	0.979	0.652-1.992	0.551-1.738
Tumor size (>20 mm *vs.* ≤20 mm)	0.308	0.367	0.771	0.794	0.468-1.271	0.481-1.311
Grade (>1 *vs.* =1)	0.066	0.085	0.563	1.720	0.305-1.040	0.928-3.188
Grade (=3 *vs.* ≤2)	0.001	0.004	3.036	2.688	1.572-5.864	1.371-5.273
TNM (>1 *vs.* =1)	0.000	0.000	3.164	2.769	1.918-5.218	1.705-4.498
TNM (=3 *vs.* ≤2)	0.000	0.001	4.000	3.565	1.987-8.055	1.734-7.332
Ki67 (≤35 *vs.* > 35)	0.001	0.004	2.956	2.597	1.585-5.512	1.362-4.950

Finally, SBEM and other clinical risk factors, including grade, disease stage, lymph node status and Ki67, were combined to determine the associations by multivariate analysis. Table 7 showed that HR for SBEM adjusted for the other risk factors remained unaffected and significant (HR = 3.370 with *p* = 0.008 for DFS and HR = 4.185 with *p* = 0.004 for OS).

**Table 7:** Multivariate analysis results of SBEM with other risk factors on DFS and OS

**Covariate**	**DFS**			**OS**		
	***P *value**	**Hazard radio**	**95% Confidence interval**	***P *value**	**Hazard radio**	**95% Confidence interval**
SBEM (3+ *vs.* <3+)	0.008	3.370	1.382-8.218	0.004	4.185	1.587-11.039
Lymph Node (Pos. *vs. *Neg.)	0.751	1.195	0.398-3.585	0.198	2.097	0.680-6.469
Grade (=3 *vs* ≤2)	0.934	1.054	0.306-3.630	0.473	1.519	0.486-4.750
TNM (>1 *vs.* =1)	0.489	1.441	0.513-4.050	0.006	3.309	1.421-7.708
TNM (=3 *vs.* ≤2)	0.001	3.851	1.744-8.500	0.015	3.529	1.274-9.772
Ki67 (≤35 *vs.* >35)	0.152	2.022	0.771-5.301	0.917	1.065	0.327-3.463

## Discussion

Breast cancer is pushed into first place in the United States and many other parts of world. Breast cancer alone is expected to account for 29% (226,870) of all new cancer cases among women [15]. Although incidence rate of breast cancer remains relatively stable in recent 5 years, its death rate declines by 34% because of the development of diagnosis and targeting medication. TNBC accounts for about 15% of all breast cancers. Patients with TNBC are more likely to experience death and distant recurrence compared to those with other cancers, and the median time to death/distant recurrence is significantly shortened. TNBC is one of solid tumors which are sensitive to chemotherapy, but other modalities, such as endocrine and targeted therapy, are not applicable. So, it is crucial to find specific markers to detect micro metastases and provide useful information to guide early therapeutic methods of TNBC patients.

Although various biological markers had been proposed for the detection of breast cancer cells, they were often affected by tumor differentiation, lower specificity and detection rate. Cyclin D1 was an effective marker for the differential diagnosis of other papillary lesions. Because Cyclin D1expressed in both lesions, it could not be used to distinguish between papilloma and papillary carcinoma lesions [16]. Petra Barros et al. found that the expression of β1 integrin had an impact in disease-specific survival (number of months from diagnosis to the time of death due to breast cancer) and could be a marker of poor prognosis in breast cancer [17]. But, β1 integrin played a role in predicting the clinical course and prognosis of several types of cancers [18], especially abundant expressing in Non-small-cell lung carcinoma [19]. By the same token, carcinoembryonic antigen (CEA) was not a specific marker of breast cancer because it was expressed at high levels in a variety of human tissues including lung, breast, and colorectal cancer. Although BRCA1 was associated with the genesis, progression, and prognosis of young breast cancer patients [20], they only accounted for about 5% of breast cancer occurrences [21]. So, researchers are searching for more promising genes to improve the screen, diagnosis and prognosis predicting of breast cancer.

SBEM was tissue-specific protein and only expresses in mammary and salivary glands [7]. SBEM could serve as a useful marker for breast nodal metastasis, and for detection of micro metastatic cells within lymph nodes. Also, it was used for the differential diagnosis of the primary origin of an unknown metastasis, especially in high grade and ER/PR-negative tumors [7]. Hinde et al. confirmed that the predictive power of IHC criteria appeared to be similar to that of gene expression analysis. The IHC information could be used to improve therapeutic decisions, mainly for luminal B, Her2- over-expressing and basal-like subtypes [22]. So, we examined SBEM levels in FFPE tissue sections by IHC test and then analyzed the correlation of SBEM expression with DFS and OS. Liu et al. reported that SBEM protein expression correlated with tumor size, TNM staging and lymph node metastasis [9]. Ki67 is used to assess the prognosis of cancer patients [23]. It would be indicated that SBEM were related to prognostic value with Ki67. Overall these risk factors, including age, grade, size, disease stage, lymph node status, and Ki67, were analyzed in our study. Our data showed that the detection rate of SBEM in FFPE tissue of TNBC was 58%, which was higher than previous report [8]. SBEM expression levels positively correlated with DFS and OS in TNBC patients. The Cox’s proportional hazards regression model showed that SBEM was independent for grade, age, disease stage, lymph node status, and Ki67. When we adjusted SBEM to combine with each clinical risk factor, SBEM expression still remained significant. Multivariate analysis showed that patients with a high SBEM expression of 3+ represented a higher risk of recurrence and mortality than those with a low SBEM expression (HR = 3.370 with *p* = 0.008 for DFS and HR = 4.185 with *p* = 0.004 for OS). SBEM could be regarded as an independent prognostic factor in TNBC.

We believed that SBEM would show much more advantages than other protein–based biomarkers and would be used as prognostic indicator. Meanwhile, SBEM expression in PB (Peripheral blood) of breast cancer patients was markedly higher than that of healthy donors and other cancer patients [9]. Determination of SBEM protein in tissue and mRNA expression in PB of TNBC patients maybe helpful for early diagnosis, choice of treatment, decision of the degree of malignancy and risk prediction of recurrence. However, it is necessary to determine the function of a certain gene by carrying out large sample studies or a large meta-analysis in different institutions and hospitals. In the past decade, the findings about the relationship between Catechol-O-methyltransferase Val158Met (COMT Val108/158Met) polymorphism and breast cancer risk were inconsistent. A large meta-analysis conducted by Xue Qin confirmed that COMT Val108/158Met polymorphism may not be associated with breast cancer risk [24]. Similarly, the prognostic significance of SBEM needs further evidence in TNBC patients.

Due to different breast cancer subtype are associated with different gene expression patterns, it is significant to identify the particular gene to suit the proper biological characteristic of a certain type of primary tumor. SBEM over-expression maybe the special characteristic of tumor cells in TNBC. In conclusion, we have done some really nice research in which SBEM shows its prognostic value in TNBC. Our findings may eventually lead to wide application of SBEM as a tumor marker or a target gene for therapy and rapid development in the diagnostic and therapeutic products for TNBC patients in future.

## Conclusion

We have demonstrated that SBEM is differentially expressed in TNBC patients by immunostaining and SBEM may be best viewed as an independent prognostic factor of DFS and OS. The expression of SBEM does significantly correlate with a DFS and an OS of TNBC patients. We suggest that SBEM could be a promising prognostic biomarker in TNBC patients for cancer diagnostics, as well as be a possible target for the treatment of TNBC patients.

## Consent

Written informed consent was obtained from the patient for publication of this report and any accompanying images.

## Abbreviations

SBEM: Small breast epithelial mucin; TNBC: Triple-negative breast cancer; ER: Estrogen receptor; PR: Progesterone receptor; HER2: Human epidermal growth factor receptor-2; OS: Overall survival; DFS: Disease-free survival; IHC: IHC; PB: Peripheral blood; RT–PCR: Reverse transcription polymerase chain reaction; TNM: Tumor node metastasis; FISH: Fluorescence in situ hybridization; NCCN: National comprehensive cancer network; CEA: Carcinoembryonic antigen

## Competing interests

The authors declare that they have no competing interests.

## Authors’ contributions

LL contributed to the study design, data acquisition and analysis and drafted the manuscript; ZL was involved in data acquisition and revision of the manuscript; SQ, ZZ and YL worked on aspects of data acquisition and analysis; XX contributed to the study design and data-analysis, developed the algorithm and coordinated the study; FS conceived and coordinated the study. All authors read and approved the final manuscript.
